# Role of Pigtail Catheter Drainage Versus Percutaneous Needle Aspiration in the Management of Liver Abscess: A Retrospective Analysis

**DOI:** 10.7759/cureus.20528

**Published:** 2021-12-20

**Authors:** Saurabh Kumar, Naresh K Midha, Kamlesh Ahari, Deepak Kumar, Maya Gopalakrishnan, Bharat Kumar, Gopal K Bohra, Pawan Garg, Binit Sureka, Mahendra Kumar Garg

**Affiliations:** 1 General Medicine, All India Institute of Medical Sciences, Jodhpur, IND; 2 Diagnostic and Interventional Radiology, All India Institute of Medical Sciences, Jodhpur, IND

**Keywords:** pyogenic, amoebic, pigtail catheter drainage, percutaneous needle aspiration, liver abscess

## Abstract

Introduction

A liver abscess is an important health concern in tropical countries. Effective management of liver abscesses includes appropriate antibiotics and drainage of the abscess cavity. Percutaneous abscess drainage by pigtail catheterization is now gaining popularity. We analyzed the role of pigtail catheter drainage over percutaneous aspiration in the treatment of liver abscesses.

Methods and material

This was a retrospective analytical study conducted in a tertiary care center in western India. Patients of age ≥ 18 years admitted with the diagnosis of liver abscess were included in this study. To find the effectiveness of different treatment modalities, data were analyzed in three groups: Group A (Conservative treatment), Group B (Percutaneous needle aspiration), and Group C (Pigtail catheter drainage).

Results

A total of 64 patients with a liver abscess were analyzed. There was male predominance (93.75%). Mean abscess volume in Group C (307.9 ± 212.8 ml) was significantly higher when compared to Group A (130.8 ± 72.9 ml, p = 0.03) and Group B (177.2 ± 129.5; p = 0.024). The duration of hospital stay and residual abscess volume at the time of discharge did not show a statistically significant difference between treatment groups. Pigtail catheterization of abscesses with volume >150 ml shortened the hospital stay, whereas it prolonged the hospital stay in patients with abscess volume <150 ml.

Conclusion

Percutaneous pigtail catheterization would be an operative decision for the management of liver abscess. We concluded that the use of pigtail catheterization of patients with abscess volume > 150 ml improved the clinical outcome.

## Introduction

A liver abscess is a pus-filled cavity that occurs due to the incursion of microorganisms either from hematogenous spread or by way of the biliary ductal system. The common etiology of a liver abscess includes amoebic or pyogenic and sometimes mixed infections. In the developed world, a polymicrobial pyogenic abscess is common while amoebic etiology is more prevalent in tropical countries. Despite the improvement in sanitation and the advancement of treatment modalities, amoebic and pyogenic liver abscesses are considered an important cause of morbidity or mortality in the tropical and subtropical areas of the world [1].

The major approach for the treatment of a liver abscess is antimicrobial therapy with or without radiology-guided intervention. About one-fifth of patients with liver abscesses remain refractory to antimicrobial therapy [2]. Nowadays, the generous use of sonography and computerized tomography scanning of the abdomen led to the early diagnosis and treatment of liver abscesses.

Pigtail catheter drainage (PCD) and percutaneous needle aspiration (PNA) of abscesses are proven to be more useful in large cavity size abscesses, which are refractory to conservative antimicrobial therapy [3]. Available data suggest the trend towards the preferable use of pigtail catheterization for liver abscess management [4]. Data regarding decision-making based on abscess volume and cavity size are limited. This study focused on the role of PCD with regards to the volume of abscess at hospitalization and discharge and the need for antimicrobial therapy in the management of liver abscesses.

This article was previously posted to the Research Square preprint server on July 06, 2021 (DOI: 10.21203/rs.3.rs-576254/v1).

## Materials and methods

This was a retrospective analytical study performed at a tertiary care center in western India. The study duration was July 2018 to October 2020. A total of 64 patients with a confirmed diagnosis of liver abscess were taken from the computerized patient management system of our institute.

Patients admitted with clinical features and ultrasound abdomen findings consistent with liver abscess were included in the study. Patients with age < 18 years and who refused invasive intervention were excluded from the study. Data regarding clinical features, possible risk factors, comorbidities, laboratory investigations, treatment strategies, and outcomes were collected in predesigned proforma. Patients with positive *Entamoeba histolytica* serology and/or positive stool microscopy for amoebic trophozoites and cysts are considered as of amoebic etiology. Positive pus culture and/or blood culture for bacteria was considered as of pyogenic origin. Mixed etiology (amoebic and pyogenic) was considered if both were positive.

All patients were started on empirical intravenous ceftriaxone (1 gm bid) and metronidazole (500 mg tid) during hospitalization. Antibiotics were modified according to culture sensitivity and if there was no response to initial therapy. Patients were allocated into three groups based on treatment modalities for further analysis. Group A consisted of patients who received conservative management in the form of intravenous antibiotics only, Group B included patients who underwent percutaneous needle aspiration (PNA), and Group C included those who underwent pigtail catheter drainage (PCD). All patients were discharged with recovery, so the duration of hospital stay in days was taken for outcome analysis.

Statistical analysis

Data were analyzed by using SPSS version 20 (IBM Corp., Armonk, NY). Continuous variables were represented as mean (± standard deviation); numbers and percentages were used for categorical variables. The analysis of variance (ANOVA) test was used to find the statistical significance of continuous variables between groups. Pearson's coefficient correlation and multiple logistic regression were used to find an association between variables.

## Results

A total of 64 patients with a liver abscess were analyzed. The mean age of the study population was 43.8 ± 15.3 years with male predominance (93.75%). Alcohol and smoking were the most common associated risk factors. Pain in the abdomen was the most common presenting complaint (84%), followed by fever (78%). Right hypochondrium tenderness was the most common finding on per-abdominal examination (Table 1).

**Table 1:** Demography, etiology, and clinical profile of patients with a liver abscess

Total patients (n=64)	Parameters	Number (Percentage)
Gender	Male	60 (93.75%)
	Female	4 (6.25%)
Risk Factors	Alcoholic	39 (61%)
	Smoking	38 (59.4%)
	Diabetic	6 (9.4%)
	Hypertension	4 (6.25%)
Etiology	Amoebic	50 (78%)
	Pyogenic	4 (6.25%)
	Mixed	3 (4.7%)
Symptoms	Pain abdomen	54 (84.3%)
	Fever	50 (78%)
	Anorexia	40 (62.5%)
	Nausea/vomiting	26 (40.6%)
	Weight loss	26 (40.6%)
Signs	Pallor	5 (7.8%)
	Icterus	5 (7.8%)
	Ascites	8 (12.5%)
	Pleural effusion	19 (29.7%)

The right lobe abscess was predominant (82.5%), followed by bi-lobar involvement (9.4%). Multiple liver abscesses were found in 12% of patients. Etiology could be ascertained in 57 (89%) patients (78% amoebic, 6.3% pyogenic, and 4.7% mixed amoebic and pyogenic) while it was not evident in seven (11%) patients, by amoebic serology, or by pus culture.

All patients received antibiotic therapy. Twelve (18.8%) patients were treated with PNA, and 35 (54.7%) patients underwent pigtail drainage; the decision of the need for intervention was made by the treating team, including the clinician and interventional radiologist.

Demographic, laboratory, and management data were analyzed among three treatment groups (Table 2). Mean abscess volume in Group C (307.9 ± 212.8 ml) was significantly higher when compared to Group A (130.8 ± 72.9 ml; p=0.03) and Group B (177.2 ± 129.5; p=0.024), while there was no significant difference between Group A and Group B (p=0.27). The duration of hospital stay and residual volume at the time of discharge did not differ significantly between treatment groups (Table 2).

**Table 2:** Comparative analysis of data between treatment strategies groups Hb- Hemoglobin, TLC- Total leukocyte count, ESR- Erythrocyte sedimentation rate, HsCRP- Highly sensitive C-reactive protein, AST- Aspartate aminotransferase, ALT- Alanine aminotransferase

Variables (Mean±SD)	Total (n=64)	Group A (n=17)	Group B (n=12)	Group C (n=35)	P value
Age (Years)	44±15.3	40.1±12.9	44.8±20.1	45.5±14.4	0.51
Duration of Hospital Stay (Days)	17.8±10.6	14.4±13.7	16.7±8.4	19.8 ± 9.3	0.23
Hb (gm%)	11.7 ± 2.0	13.0±2.0	11.8±1.6	11.0±1.8	0.003
TLC	14133±6197	14036±6309	11267±6072	15275±6006	0.14
ESR	77.6±27.9	74.6±26.5	68.4±24.7	83.1±29.4	0.25
HsCRP	127.1±84.3	130.3±107.8	126.3±64.9	125.8±80.2	0.98
AST	59.1±49.6	35.4±18.1	56.2±42.9	71.4±59.2	0.13
ALT	51.1±41	39.1±34.4	62.1±46.1	52.6±41.5	0.31
Total Bilirubin	1.2±0.9	0.9±0.6	1.2±0.9	1.4±1.1	0.21
Abscess Volume on day of admission	231.5±192.1	130.8±72.9	177.2±129.5	307.9±212.8	0.001
Abscess Volume on day of Discharge	19.9±16	16.5±16.6	15.8±14.9	22.9±15.9	0.26
Duration of Metronidazole	23.1±8	20.4±6.3	23.4±7.5	24.3±8.7	0.29

**Figure 1:**
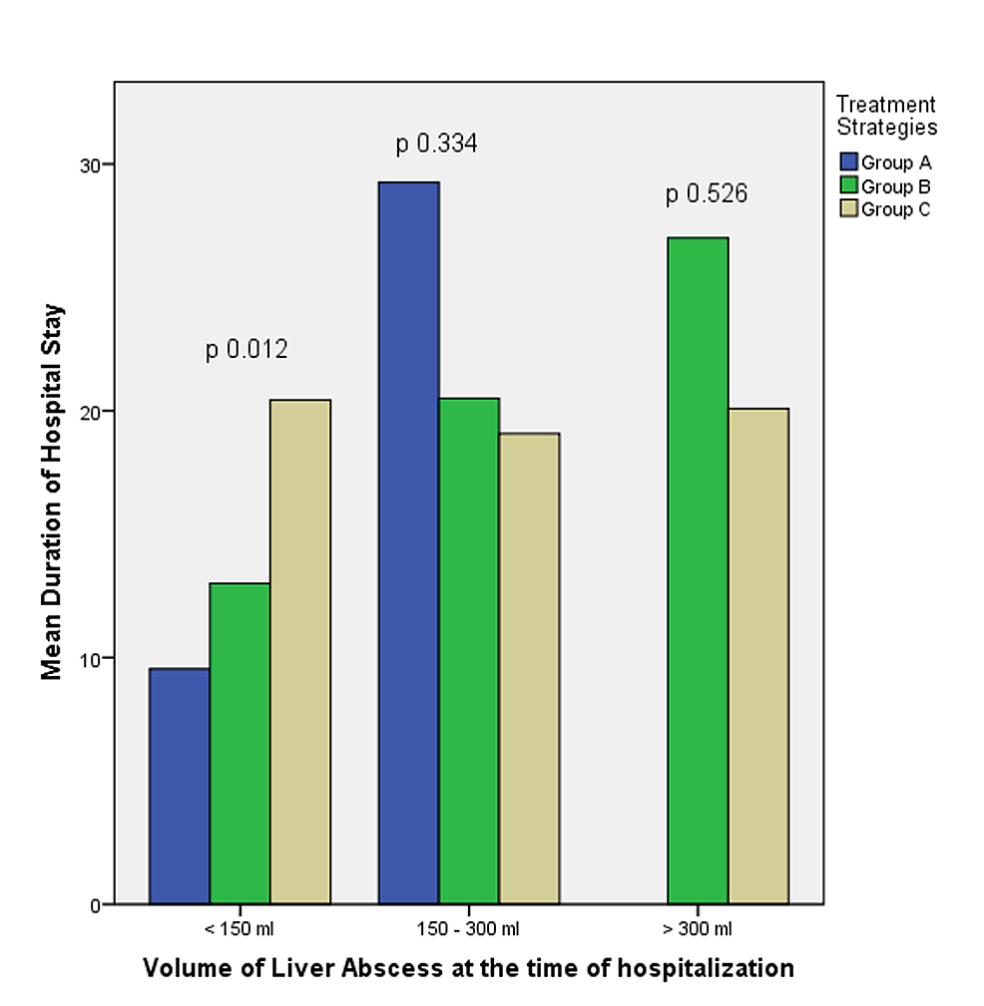
Association between duration of hospitalization and treatment strategies according to liver abscess volume

The association between the duration of hospital stay and treatment strategies was further analyzed according to liver abscess volume at the time of hospitalization (Figure 1). This showed that PCD in abscess volume < 150 ml was associated with a statistically significant increase in the duration of hospital stay (p = 0.012). However, PCD between an abscess volume of 150-300 ml was not associated with an increase in the duration of hospital stay (Figure 1).

The involvement of the right lobe and amoebic etiology were found comparable in all three groups (Table 3). The duration of hospital stay was positively correlated with the duration of fever (r = 0.28, p = 0.028) and total leucocyte count at the time of hospitalization (r = 0.35, p = 0.003).

**Table 3:** Distribution of amoebic etiology and right lobe involvement in different treatment strategies

Variables	Group A (n=17)	Group B (n=12)	Group C (n=35)	P-value
Amoebic Etiology	13 (76%)	10 (83%)	27 (77%)	0.19
Right Lobe Involvement	12 (70%)	10 (83%)	29 (82%)	0.65

## Discussion

A liver abscess is an imperative health issue in tropical countries. The common etiology of liver abscesses are *E. histolytica *(amoebic), bacterial (pyogenic), and *Mycobacterium tuberculosis *[5]. An amoebic liver abscess is more common in tropical countries and their incidence is >50 million cases and 1,00,000 deaths per year [6-7]. The involvement of the right lobe was predominant (82.5%) in this study, which was like previous studies [5,8]. Etiology could be ascertained in 89% of cases, of which 78% were of amoebic etiology. The disease is more common in the younger population, so effective treatment is required for a decrease in morbidity and mortality in the productive age group. Common presenting complaints of liver abscess are abdominal pain, fever, loss of appetite, and weight loss [9]. With the wide availability of ultrasound, the diagnosis of liver abscess becomes easier but effective treatment with judicious selection of antimicrobial and early source control is still an area of debate. With the advent of interventional radiology, percutaneous treatment in the form of either PNA or PCD is preferred in the management of liver abscesses [4].

The mean cavity volume was significantly higher in Group C (PCD group) when compared to Groups A and B in the present study. Despite this, Group C had a comparable duration of hospital stay and duration of antibiotic therapy. Few important randomized control trials have been conducted to compare the efficacy between PCD and PNA [1,10-14] with variable results. Among them, three trials showed PCD as the preferred method for abscess drainage, and it is more effective if cavity size is > 10 cm [1,10,13]. Yu et al. concluded that there was no significant difference in the hospital stay and clinical outcomes when compared PCD vs PNA in abscess cavity size of around 5 cms [11]. However, Zerem and Hadzic et al. concluded that PNA was the preferred method if the cavity size is smaller than 5 cm [12]. A metanalysis by Cai YL et al. also favored PCD as a preferred method of management and reported a lower success rate with PNA [4]. The success rate of PNA is considered low due to the need for multiple attempts at aspiration in larger cavity size abscesses and the risk of re-accumulation. We also analyzed the effect of abscess volume and treatment strategies on the duration of hospital stay. PCD was found to be an effective choice of abscess drainage if the volume was >150 ml. However, it was associated with higher duration of hospital stay if used in abscess volumes <150 ml. Kulhari M et al. reported better clinical outcomes with PCD over PNA in the patients with approximately similar volumes of liver abscess (293.2±130.3 mL in the PCD group and 291.4±138.8 mL in the PNA group, P = 0.925) [14]. Rajak et al. also showed that higher abscess volume was associated with PNA failure [10].

Various studies described the comparison between conservative treatment and the percutaneous aspiration approach. Most of these studies were conducted before the widespread use of PCD for the treatment of liver abscesses. Results of these studies showed that PNA was more useful in higher abscess volume [15-17]. In this study, conservative management was non-inferior to PNA for the duration of hospitalization and duration of antibiotics used even when abscess volume was similar in both groups (130.8±72.9 vs 177.2±129.5 ml, p = 0.27).

PCD-related complications were major issues in previous reports [17]. There were no significant complications found due to PCD in this study. The recent studies also favor that complication rates were not significantly different in PCD vs PNA in the management of liver abscesses [18], while PCD reduces cavity size and abscess volume faster and is associated with fewer complications than PNA.

Lobe involvement and etiology were not found to affect the outcome of different treatment strategies in our study.

The limitations of this study were retrospective analysis and selection bias regarding the preferable use of pigtail catheterization in patients with large abscess volumes

## Conclusions

Liver abscess is mostly a disease of young and middle-aged males in tropical countries. Effective management with drainage decreases the duration of antibiotics and hospital stay. With the advent of interventional radiodiagnosis, percutaneous PCD became a favored decision for the management of liver abscesses. We concluded that the use of pigtail catheterization, as well as percutaneous needle aspiration, in patients with abscess volume >150 ml improves the clinical outcome and reduces the disease-related morbidity. Pigtail catheterization is found as a better intervention modality than percutaneous needle aspiration in patients with an abscess volume of more than 300 ml. Large RCTs with a definite protocol will be required in the future for effective guidelines on the management of liver abscess.
